# Evaluating the local expression pattern of IGF-1R in tumor tissues and the circulating levels of IGF-1, IGFBP-1, and IGFBP-3 in the blood of patients with different primary bone tumors

**DOI:** 10.3389/fonc.2022.1096438

**Published:** 2023-01-13

**Authors:** Mohammad Amin Vaezi, Amir Reza Eghtedari, Banafsheh Safizadeh, Pegah Babaheidarian, Vahid Salimi, Fatemeh Adjaminezhad-Fard, Sahar Yarahmadi, Alireza Mirzaei, Mahtab Rahbar, Masoumeh Tavakoli-Yaraki

**Affiliations:** ^1^ Department of Biochemistry, School of Medicine, Iran University of Medical Sciences, Tehran, Iran; ^2^ Department of Pathology, School of Medicine, Iran University of Medical Sciences, Tehran, Iran; ^3^ Department of Virology, School of Public Health, Tehran University of Medical Sciences, Tehran, Iran; ^4^ Bone and Joint Reconstruction Research Center, Shafa Orthopedic Hospital, Iran University of Medical Sciences, Tehran, Iran

**Keywords:** insulin-like growth factor-1 (IGF-1), bone tumors, osteosarcoma, Ewing sarcoma, giant cell tumors, tumor recurrence

## Abstract

**Introduction:**

The present study tried to provide insights into the expression pattern and diagnostic significance of the IGF-1 axis main mediators in three main primary bone tumor types with different degrees of severity.

**Methods:**

The real-time qRT-PCR (to analyze IGF-1R gene expression), the immunohistochemistry (to measure IGF-1R protein), and the ELISA assay (to assess the circulating level of IGF-1, IGFBP-1, and IGFBP-3) were applied to confirm this hypothesis. A total number of 180 bone tissues (90 tumors and 90 noncancerous adjacent tissues) and 120 blood samples drained from 90 patients with bone tumors and 30 healthy controls were enrolled in the study. The association of insulin-like growth factor (IGF)-1 axis expression pattern with the patient’s clinical pathological characteristics and tumor aggressive features, the diagnostic and predictive values were assessed for all tumor groups.

**Results:**

A significantly elevated level of IGF-1R gene and protein was detected in bone tumors compared to the noncancerous bone tissues that were prominent in osteosarcoma and Ewing sarcoma compared to the GCT group. The positive association of the IGF-1R gene and protein level with tumor grade, metastasis, and recurrence was detected in the osteosarcoma and Ewing sarcoma groups. The circulating level of IGF-1, IGFPB-1, and IGFBP-3 were increased in osteosarcoma and Ewing sarcoma and GCT groups that were correlated significantly to the tumor severity. The ability of the IGF-1 axis to discriminate between bone tumors also malignant and benign tumors was considerable.

**Discussion:**

In summary, our data suggested that IGF-1R, IGF-1, IGFBP-1, and IGFBP-3 levels are associated with bone tumor malignancy, metastasis, and recurrence that might serve as biomarkers for osteosarcoma and Ewing sarcoma recurrence.

## 1 Introduction

The insulin-like growth factor (IGF) family is a multifunctional system composed of polypeptide hormones (IGF-1 and 2) and their surface receptors; Insulin-like growth factor receptors I and II (IGF-1R, IGF-2R) and several IGF-binding proteins (IGFBPs) (1). As a tyrosine kinase receptor, the IGF receptor is a homolog of the insulin receptor (IR) with the highest affinity for IGF, which upon activation, mediates tyrosine phosphorylation and activates the Shc-dependent downstream signaling pathways (2). IGF-1 as a hormone can act through a growth hormone (GH) dependent pathway and promote cell growth and proliferation (3). The half-life of IGF is mediated by IGF-binding proteins, which can primarily sequester circulating IGFs and antagonize IGF receptors; although they exert considerable functions independent of IGFs (4). Several influential metabolic functions have been postulated for IGF-1, including its stimulatory role in protein synthesis and fatty acid utilization, as well as its regulatory role in GH and insulin secretion, which can provide sufficient signals for growth (5). However; The critical role of the IGF family in the context of carcinogenesis through both canonical and non-canonical IGF receptor signaling and interaction with multiple signaling pathways has become evident (6). Activation of IGF-1R has been shown to recruit anchor proteins such as Shc-transforming protein 1 (Shc), which mediates mitogen-activated protein kinase (MAPK) activation *via* Shc-dependent downstream signaling pathways (2). Also, IGF-1R activation can recruit the insulin receptor substrate (IRS), resulting in activation of the PI3K/Akt pathway, which overall favors cell cycle progression, intensified proliferation, increased mitogenesis, and resistance to apoptosis (7). In addition, IGF-1R translocation to the nucleus mediates IGF-1R binding to the enhancer region and triggers transcription of target genes such as cyclin D1, leading to IGF-1R-mediated cell transformation. IGF-1R was shown to mediate E-cadherin cleavage from the cytoskeleton and increase β-catenin levels, which may facilitate cell motility and invasion (8). Because of the multiple effects of the IGF family on cancer cell fate, altering the expression levels of IGF axis mediators and their function in cancer seems reasonable (9). In addition to the importance of this axis for cell growth and proliferation, its key role in bone homeostasis is also of interest. It is well documented that IGFs regulate bone length, bone growth, and bone properties through their effects on chondrocytes, osteoblast, and osteoclast functions (10). It was shown that binding of IGF-1 to the IGF-1R induces receptor phosphorylation and activation of downstream insulin receptor substrate (IRS) and Shc leading to the activation of phosphatidylinositol 3 kinases (PI3K), the extracellular signal-regulated kinase (ERK)/mitogen-activated protein kinase (MAPK), AKT and mTOR signaling pathways (11, 12). Besides the fact that these signaling pathways are involved in controlling metabolic process during cell differentiation, it was shown that the phosphorylated AKT activate substrates such as mTORC1, Forkhead transcription factor (FoxO) and glycogen synthase kinase3α,β (GSK3) that regulate bone cell proliferation, differentiation and skeletal development (13–15). In addition, it appears that the interaction of IGFs with steroid hormones and parathyroid hormones plays a role in bone homeostasis (16). Bone sarcomas are tumors of mesenchymal origin with unresolved challenges such as complicated diagnosis due to morphological overlap with bone lesions, inefficient response to chemotherapeutic approaches, high rate of metastasis, and the possibility of tumor recurrence (17). Efforts are therefore being made to develop plausible therapeutic approaches and effective diagnostic biomarkers for the early detection of bone tumors (18). However, the relevance of the IGF family for the pathogenesis of bone tumors is exploited in some studies. For example; The high proliferative activity of cells of the human osteosarcoma cell line HOS 58 was shown to be associated with increased IGF-1R expression, indicating the regulatory role of the IGF axis in bone cancer proliferation and differentiation (19). The current study is designed to shed more light on the expression profile of the IGF-1 axis in predominant bone tumor types and to underscore the status of IGF-1, its receptor, and major binding proteins in bone tumor severity.

## 2 Materials and methods

### 2.1 Patients and sample collection

A total number of 180 bone tissues (30 osteosarcoma tumors and 30 paired noncancerous tissue, 30 Ewing sarcoma tumor and 30 paired noncancerous tissue, and 30 giant cell tumors (GCT) and 30 paired noncancerous tissue) were studied in the current study. The sample collection, preparation, molecular and serological assessments were conducted following the declaration of Helsinki with local ethical approval (20) and informed consent and regulations of our institute’s ethical standards. The patient’s demographic features are described in our previous study (21) and a brief description of the number of patients in each tumor group in terms of gender, age distribution, tumor grade, metastasis, recurrent, and receiving or not receiving chemotherapy treatment is summarized in **Supplementary Table 1**. The age distribution of the patients with osteosarcoma tumors was as 13.33% of them were between 15-20 years of age; 23.33% of them were between 20-30 and 63.33% were over 30 years of age. The average age of patients with osteosarcoma tumors was 37.76± 3.17 years and 53.3% of them were male. The age distribution of the patients with Ewing sarcoma tumors was 36.66% between 15-20 years of age; 30% between 20-30, and 33.33% were over 30 years of age. The average age of patients with Ewing sarcoma was 25.60 ± 1.64 years, and 40% of them were male. Regarding patients with GCT, 26.66% of patients were between 15-20 years of age; 26.66% were between 20-30 and 46.66% were over 30 years of age. The average age of patients with GCT was 31.50 ± 2.8 and 56.7% of them were male. To check the serum level of the investigated factors, 6 ml of blood was collected from each patient, and therefore 30 blood samples from the group of patients with osteosarcoma tumor, 30 blood samples from the group of patients with Ewing sarcoma and 30 blood samples from the group of patients with GCT was received and subjected to serum separation and further assays. To compare the changes in the serum levels of the investigated factors in the patient group, 30 blood samples were collected from healthy individuals who matched with the patients in terms of age and gender and were analyzed. The age distribution of the healthy controls was 16.66% between 15-20 years of age; while 33.33% between 20-30 and 50% were over 30 years of age and the average age was 34.59 ± 3.02. An equal number of males and females were selected among healthy individuals. Blood samples were obtained from each patient immediately before surgery and in fasting conditions, and for a better comparison, blood samples from healthy individuals were also obtained in similar conditions. A pair of tumor and marginal tissue samples were taken from all the patients who were subjected to surgery at Shafa Orthopedic Hospital and were kept immediately at -80 for later assays (22). In this study, patients with different disease satges and tumor grades were examined. The grading of bone tumors is based on the degree of cellularity and cell differentiation which is a valuable prognostic indicator for patients with primary bone tumors (23, 24). Accordingly and based on the pathology reports in this study and our previous reports (18, 21), tumors were divided based on the degree of differentiation into low (well-differentiated) and high (low-differentiated) grades. Also based on the patient’s pathology information, if the primary bone tumor of the patient had metastasized to distant organs, this tumor was included in the metastatic tumor category and if no evidence of distant metastases was detected in the patient, the tumor was included in the non-metastatic tumor group. This study collected the primary bone tumor from both metastatic and non-metastatic tumors. Also, a local recurrent bone tumor indicates tumor returns one or more years after the patient finishes an initial treatment. Additionally, patients who received a course of a chemotherapy treatment at least 1 month before surgery were considered as patients receiving chemotherapy regimen in the current study. In terms of tumor characteristics in patients with osteosarcoma tumors, 63.3% of tumors were high grade, 30% of tumors were metastatic and 26.7% of tumors were recurrent. Moreover, 46.7% of patients with osteosarcoma had tumor sizes over 10 cm and 50% of patients received chemotherapy before surgery. As previously described (21), for patients with osteosarcoma, the combination of doxorubicin, cisplatin, and methotrexate was applied as a chemotherapy treatment protocol. Amongst patients with Ewing sarcoma, 66.7% of patients had a high-grade tumor, 70% had a non-metastatic tumor, 76.7% had a non-recurrent tumor, 40% had a big tumor size and 60% received a chemotherapy regimen. For patients with Ewing sarcoma, the combination of vincristine, cyclophosphamide, and doxorubicin was used for chemotherapy treatment. No report of metastasis, tumor recurrent, and high grade tumor was received for GCT patients enrolled in this study.

### 2.2 RNA extraction, cDNA synthesis, and real-time PCR

To determine the IGF-1R gene expression level, total RNA was extracted from fresh tumor and healthy bone tissue of each patient using Trizol (Invitrogen, Grand Island, USA) following the instructions. The Nanodrop spectrophotometer (Nanodrop Technologies) and electrophoresis using 1% agarose gel were applied for determining the quantity and integrity of the extracted RNA from each patient. The PrimeScript First Strand cDNA Synthesis Kit (Takara, Japan) was used for cDNA synthesis from RNA (1 µg) of each sample and the SYBR Premix Ex Taq II (Takara, Japan) was utilized for quantification of mRNA level using Applied Biosystems Step One Plus, Real-Time System (Applied Biosystems, USA). To normalize the IGF-1R expression, the β-actin expression level was simultaneously assessed for all samples, and the reaction running program was as: 10 minutes (95°C), 5 seconds (95°C, 40x), 20 seconds (55°C), and 35 seconds (60°C). The primer sequences were as follows: IGF-1R forward primer: 5’– GGAACCTGAGAATCCCAATG -3’, IGF-1R reverse primer: 5’- GAGAGATGTGGCCTGAATC -3’ (Tm=57), β-actin forward primer: 5’-GAT CTC CTT CTG CAT CCT GT-3’, β-actin reverse primer: 5’-TGG GCA TCC ACG AAA CTA C- 3’ (Tm=57). The PCR product length was evaluated using electrophoresis and the primer specificity was confirmed by evaluating their melt curve.

### 2.3 Assessment of serum IGF-1, IGFBP-1 and IGFBP-3 levels

The IGF-1, IGFBP-1, and IGFBP-3 levels in the serum of the patients and healthy subjects were measured using ELISA assay kits according to the manufacturer’s recommendations. A human IGF-1 Quantikine ELISA Kit (R&D, USA, Cat No.# DG100B) with an analytical sensitivity of 0.022 ng/mL, a Human IGFBP-3 Quantikine ELISA Kit (R&D, USA, Cat No.# DGB300) with an analytical sensitivity of 0.14 ng/mL and a Human IGFBP-1 DuoSet ELISA kit (R&D, USA, Cat No.# DY871) with an analytical sensitivity of 31.1 pg/mL were applied in this regards.

### 2.4 Protein assessment *via* immunohistochemistry

The local protein expression level of the IGF-1 receptor was assessed in tumor and normal bone tissues *via* immunohistochemistry. The dilution of 1:100 of IGF-1R (Abcam, USA, Cat No. # ab39675) and t1:200 of the anti-rabbit IgG HRP-conjugated secondary antibody (Cell Signaling, the Netherlands, Cat No. # 7074) was applied for staining. The tissue processing, preparation, and staining protocol are based on our previously described method. Briefly, the frozen tissue blocks were prepared using Optimal Cutting Temperature (OCT) embedding medium and tissues were fixed in paraformaldehyde (4%). The tissue sections (10nm) were prepared using cryotome and incubated in Triton (3%) for 30 minutes to induce membrane permeability following appropriate washing. The non-specific antigenic sites were blocked using goat serum (10%) and the tissue slides were exposed to 1 µl of 3′-diaminobenzidine (DAB) chromogen and 20µl of DAB substrate for 10 minutes. The stained tissues were evaluated by a pathologist and the intensity of IGF-1R staining was determined using Image J software and the percentage of positive reactivity was reported. In this regard, multiple images were taken from each sample and were processed with software by converting them to black-and-white images. The percentages of cells were determined by setting up a threshold and the threshold was adjusted by removing the background signals. The IHC images were analyzed based on the threshold and evaluated at least three times in a blinded manner (25, 26). The weak intensity of IGF-1R indicates <10% immune reactivity, the moderate intensity of IGF-1R indicates 10-20% immune reactivity and the strong intensity of IGF-1R indicates > 20% immune reactivity.

### 2.5 Statistical analysis

**Figure 1:**
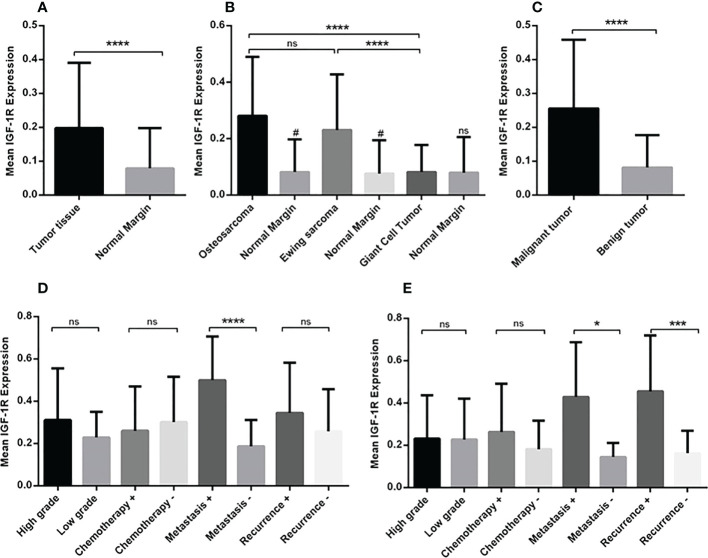
The gene expression level of IGF-1R in osteosarcoma, Ewing sarcoma and GCT. The mean mRNA expression level of IGF-1R increased in bone tumor (N=90) tissues compared to the adjacent noncancerous tissues (N=90) **(A)**. The elevated level of expression was detected in osteosarcoma (N=30), Ewing sarcoma (N=30) and GCT (N=30)compared to the margin tissues (30 tumor margin tissues that were obtained and compared for each tumor group separately) also osteosarcoma (N=30) compared to the GCT (N=30) **(B)**. The difference in the IGF-1R expression in malignant (N=60) and benign tumors (N=30) is shown **(C)**. The comparison of IGF-1R expression level in tumor subtypes including tumor grade, chemotherapy-received status, metastasis, and recurrence is shown separately for osteosarcoma **(D)** and Ewing sarcoma **(E)**. The number of examined samples in each tumor subtype is shown in **Supplementary Table 1**. The statistical differences between groups are shown as asterisks (*P <0.05, ***P <0.001, ****P<0.0001), (ns) indicates unspecific, (#) indicates P<0.0001 for comparing osteosarcoma and Ewing sarcoma with adjacent noncancerous tissues.

**Figure 2:**
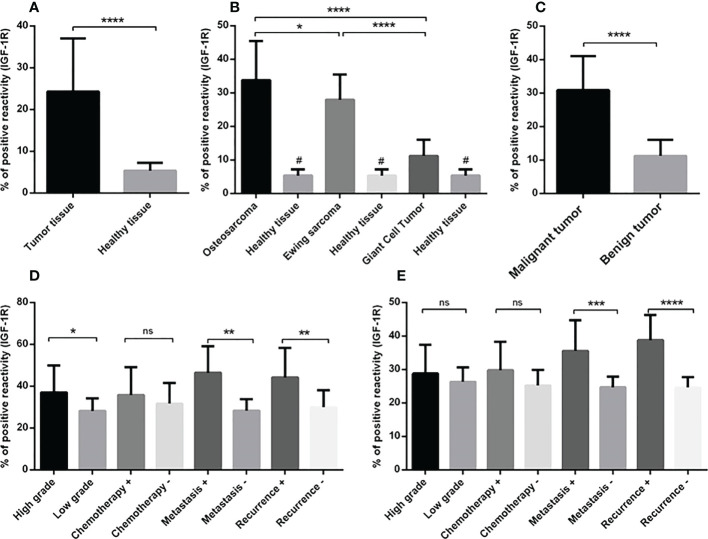
The IGF-1R protein expression level in primary bone tumors. The protein level of IGF-1R is represented as a positive reactivity percentage and the level was increased in bone tumors (N=90) versus tumor margins (N=90) **(A)**, also bone tumor subtypes versus adjacent noncancerous tissues **(B)**. The osteosarcoma tumors (N=30) expressed more IGF-1R protein compared to Ewing sarcoma (N=30) and GCT (N=30) **(B)**. The elevated level of IGF-1R protein in malignant tumors (N=60) versus benign tumors (N=30) is shown **(C)**. The elevated IGF-1R protein level is demonstrated in high-grade (N=19), metastatic (N=9) and recurrent (N=8) osteosarcoma tumors **(D)** and in metastatic (N=9) and recurrent (N=7) Ewing sarcoma tumors **(E)**. The number of examined samples in each tumor subtype is shown in **Supplementary Table 1**. The statistical differences between groups are shown as asterisks (*P <0.05, **P <0.01, ***P <0.001, ****P<0.0001) and (ns) indicates unspecific. (#) indicates P<0.0001 for comparing osteosarcoma, Ewing sarcoma, and GCT with adjacent noncancerous tissues.

To analyze the gene expression level of the IGF-1 receptor, the comparative Ct (2^-ΔCt^) method was used that is based on the subtraction of the Ct of the IGF-1 receptor from the Ct of the β-actin, as an endogenous gene for each sample (tumor and/or margin). The normal distribution of data was evaluated by Kolmogorov-Smirnov analysis and according to the result, a nonparametric Mann–Whitney U-test was applied to compare the IGF-1 receptor gene expression level between different groups (Results are illustrated in **Figure 1**, and statistical details are provided in **Supplementary Table 2**). The parametric unpaired t-test was used to analyze the IGF-1R protein level, IGF-1, IGFBP-1 and IGFBP-2 between different patient groups (Results are illustrated in **Figures 2**, **4**–**6**). The diagnostic value of the IGF-1R gene and protein, IGF-1, IGFBP-1, and IGFBP-3 levels in patients with bone cancer was calculated by the receiver operating characteristic (ROC) curves and area under the curve (AUC), also the optimal cut-off values points were defined based on Youden index (27) (Results are illustrated in **Table 1**). The association of IGF-1, IGFBP-1, IGFBP-3, and IGF-1R with demographic features of patients with bone cancer was using Spearman’s correlation coefficient test (Results are illustrated in **Table 2**). The logistic regression was used to determine the possible effect of the IGF-1 axis in tumor features prediction (Results are illustrated in **Table 3**). The mean and standard error of the mean (SEM) of IGF-1R mRNA level also IGF-1, IGFBP-1, and IGFBP-3 serum levels are reported in the results with two decimal points for each sample group, separately. The Graph Pad Prism Version 6 (Graph Pad Software, San Diego California) and Statistical Package for Social Science (SPSS v.16) was applied for statistical analysis, and P-values < 0.05 (two-tailed) were considered statistically significant.

**Table 1:** The value of IGF axis to discriminate between different groups of primary bone tumors (ROC curve information).

Variable	Groups	Cutoff point	Sensitivity (%)	Specificity (%)	AUC	P-value
**IGF-1**	Patient Vs. Control	> 173.8	95.56	100.0	0.9878	<0.0001
**IGF-1**	Malignant Vs. Control	> 252.6	98.33	100.0	0.9922	<0.0001
**IGF-1**	Benign Vs. Control	> 173.8	90.00	100.0	0.9789	<0.0001
**IGF-1**	Malignant Vs. Benign	> 378.7	76.67	80.00	0.8189	<0.0001
**IGFBP-1**	Patient Vs. Control	> 33.65	71.11	63.33	0.6869	0.0022
**IGFBP-1**	Malignant Vs. Control	> 32.34	88.33	56.67	0.7672	<0.0001
**IGFBP-1**	Benign Vs. Control	> 33.80	53.33	63.33	0.5261	0.7283
**IGFBP-1**	Malignant Vs. Benign	> 41.25	53.33	83.33	0.7272	0.0005
**IGFBP-3**	Patient Vs. Control	> 2.260	86.67	90.00	0.9335	<0.0001
**IGFBP-3**	Malignant Vs. Control	> 2.630	93.33	96.67	0.9869	<0.0001
**IGFBP-3**	Benign Vs. Control	> 2.260	63.33	90.00	0.8267	<0.0001
**IGFBP-3**	Malignant Vs. Benign	> 3.275	83.33	83.33	0.8889	<0.0001
**IGF-1R** **(Gene)**	Patient Vs. Normal margin	> 0.09488	68.89	75.56	0.7294	<0.0001
**IGF-1R** **(Gene)**	Malignant Vs. Normal margin	> 0.09488	86.67	75.00	0.8354	<0.0001
**IGF-1R** **(Gene)**	Benign Vs. Normal margin	> 0.1568	26.67	90.00	0.5117	0.8766
**IGF-1R** **(Gene)**	Malignant Vs. Benign	> 0.06304	90.00	63.33	0.8081	<0.0001
**IGF-1R** **(Protein)**	Patient Vs. Control	> 10.13	87.78	100.0	0.9435	<0.0001
**IGF-1R** **(Protein)**	Malignant Vs. Control	> 15.03	100.0	100.0	1.000	<0.0001
**IGF-1R** **(Protein)**	Benign Vs. Control	> 8.935	70.00	96.67	0.8306	<0.0001
**IGF-1R** **(Protein)**	Malignant Vs. Benign	> 19.98	100.0	100.0	1.000	<0.0001

**Table 2:** The association of IGF-1, IGFBP-1, IGFBP-3 and IGF-1R with bone cancer different features.

	Serum			Tumor (Gene expression)	Tumor (Protein expression)
Variable	IGF-1	IGFBP-1	IGFBP-3	IGF-1R	IGF-1R
Age					
Correlation	-0.004	0.183	0.108	0.075	0.030
P value	0.970	0.085	0.313	0.487	0.778
Tumor size					
Correlation	0.361**	0.272**	0.404**	0.419**	0.548**
P value	0.0001	0.010	0.0001	0.0001	0.0001
Malignancy					
Correlation	0.521**	0.371**	0.635**	0.503**	0.817**
P value	0.0001	0.0001	0.0001	0.0001	0.0001
Tumor grade					
Correlation	0.586**	0.360**	0.583**	0.309**	0.592**
P value	0.0001	0.0001	0.0001	0.003	0.0001
Metastasis					
Correlation	0.482**	0.346**	0.423**	0.562**	0.581**
P value	0.000	0.001	0.000	0.0001	0.0001
Recurrence					
Correlation	0.501**	0.393**	0.374**	0.398**	0.554**
P value	0.0001	0.0001	0.0001	0.0001	0.0001
IGF-1					
Correlation	1.000	0.360**	0.615**	0.430**	0.571**
P value		0.0001	0.0001	0.0001	0.0001
IGFBP-1					
Correlation	0.360**	1.000	0.489**	0.196	0.429**
P value	0.0001		0.0001	0.064	0.0001
IGFBP-3					
Correlation	0.615**	0.489**	1.000	0.409**	0.682**
P value	0.0001	0.0001		0.0001	0.0001
IGF-1R gene					
Correlation	0.430**	0.196	0.409**	1.000	0.561**
P value	0.0001	0.064	0.0001		0.0001
IGF-1R protein					
Correlation	0.571**	0.429**	0.682**	0.561**	1.000
P value	0.0001	0.0001	0.0001	0.0001	

**P <0.01.

**Table 3:** The regression of IGF axis (Logistic regression).

Dependent	Independent variable	OR	95% CI	P value
Malignancy (Benign Vs. Malignant)	IGF-1	0.999	0.996-1.002	0.449
	IGFBP-1	1.52	0.00-	0.999
	IGFBP-3	35.89	0.00-	0.999
	IGF-1R (Gene)	1.20	0.00-	0.996
	IGF-1R (Protein)	1.80	0.00-	0.987
Tumor grade (Low grade Vs. High grade)	IGF-1	1.00	1.00-1.01	0.050*
	IGFBP-1	1.00	0.95-1.07	0.898
	IGFBP-3	1.80	0.77-4.21	0.173
	IGF-1R (Gene)	0.34	0.01-13.47	0.568
	IGF-1R (Protein)	1.13	1.03-1.24	0.013*
Metastasis (Negative Vs. Positive)	IGF-1	1.00	1-1.01	0.412
	IGFBP-1	1.06	0.87-1.28	0.558
	IGFBP-3	0.97	0.12-8.19	0.977
	IGF-1R (Gene)	1.13	1.05-1.22	0.002*
	IGF-1R (Protein)	1.29	1.03-1.61	0.028*
Recurrence (Negative Vs. Positive)	IGF-1	1.01	1.00-1.01	0.017*
	IGFBP-1	1.09	0.98-1.22	0.115
	IGFBP-3	0.33	0.08-1.36	0.124
	IGF-1R (Gene)	1.23	0.02-98.50	0.925
	IGF-1R (Protein)	1.20	1.03-1.40	0.019*

*P <0.05.

## 3 Results

### 3.1 The IGF-1R gene expression level in osteosarcoma, Ewing sarcoma and GCT

The gene expression analysis demonstrated a significant increase in the mRNA level of IGF-1R in bone tumor tissues compared to tumor margins (P < 0.0001) (**Figure 1A**). The median of IGF-1R mRNA level was 0.1510for tumor tissues and 0.02900for tumor margins. The tumor tissues of osteosarcoma (0.2391) and Ewing sarcoma (0.1565) groups expressed higher level of IGF-1R compared to the tumor margins (0.029) (P < 0.0001); while the difference between GCT (0.03356) and margins was not notable (**Figure 1B**). Also, osteosarcoma and Ewing sarcoma tumors expressed higher level of IGF-1R compared to GCT tumors (P < 0.0001); while the difference between osteosarcoma and Ewing sarcoma tumors was not remarkable (**Figure 1B**). The malignant bone tumors (0.1999) expressed a significant gene level of IGF-1R compared to benign tumors (0.03356) (P < 0.0001) (**Figure 1C**). While comparing osteosarcoma tumors as a matter of tumor features, it was revealed that the IGF-1R mRNA level was significantly higher in metastatic tumors (0.5007) compared to non-metastatic tumors (0.1618) (P < 0.0001); however, the difference between recurrent (0.2710) and non-recurrent (0.2088) tumors was not statistically significant (**Figure 1D**). Also, no remarkable difference was detected between high grade (0.2556) compared to low grade tumors (0.2225), and chemotherapy-received tumors (0.2225) compared to their opposite counterparts (0.2864). Regarding Ewing sarcoma, both metastatic tumors (0.3280) and recurrent tumors (0.3280) expressed a significantly higher level of IGF-1R compared to non-metastatic (0.1168) (P < 0.05) and non-recurrent tumors (0.1234) (P < 0.001), respectively (**Figure 1E**). No remarkable difference was detected while comparing the high-grade (0.1492) and chemotherapy-received tumors (0.1850) to the low-grade (0.1714) and tumors with no chemotherapy history (0.1193) (**Figure 1E**). The detail of the group and intergroup comparison of IGF-1R gene expression levels in primary bone tumor tissues is illustrated in **Supplementary Table 2**.

### 3.2 The IGF-1R protein expression level in osteosarcoma, Ewing sarcoma and GCT

**Figure 3:**
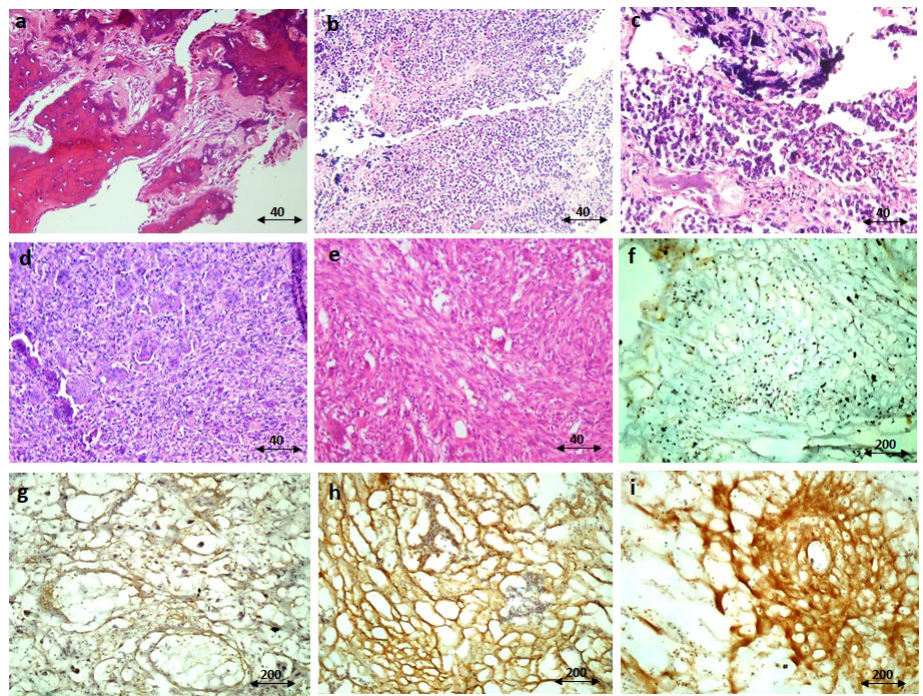
The immunohistochemistry staining of IGF-1R protein. The representative images for hematoxylin and eosin (H&E) staining of the osteosarcoma tumor tissue **(A)**, Ewing sarcoma **(B, C)**, and GCT **(D, E)** are shown. The negative immunereactivity of IGF-1R using immunohistochemistry is shown **(F)**. The weak staining intensity **(G)**, the moderate intensity **(H)**, and the strong intensity **(I)** of IGF-1R staining are shown. The scale of magnification for **(A-E)** is 40 and for **(F-I)** is 200.

The staining intensity of IGF-1R protein was assessed in tumor and margin tissues and the results are presented as the percentage of positive reactivity. As shown in **Figure 2**, bone tumors expressed a significantly higher level of IGF-1R protein compared to tumor margins (P < 0.0001) (**Figure 2A**). Moreover, the IGF-1R protein level was considerably higher in osteosarcoma tumors (P < 0.0001), Ewing sarcoma tumors (P < 0.0001) and GCT (P < 0.01) compared to their matched noncancerous tumor margins, respectively (**Figure 2B**). The IGF-1R protein over-expression was detected in osteosarcoma tumors compared to Ewing sarcoma (P=0.03) and GCT (P < 0.0001), also Ewing sarcoma tumors compared to GCT (P < 0.0001) (**Figure 2B**). The malignant bone tumors expressed more IGF-1R protein compared to benign tumors (P < 0.0001) (**Figure 2C**). The IGF-1R protein level was significantly increased in high-grade, metastatic and recurrent osteosarcoma tumors compared to low-grade (P=0.01), non-metastatic (P=0.002) and non-recurrent tumors (0.008) (**Figure 2D**). Also, the IGF-1R protein level was significantly elevated in metastatic and recurrent Ewing sarcoma tumors compered to non-metastatic (P=0.0008) and non-recurrent tumors (P < 0.0001) (**Figure 2E**). The IGF-1R protein level in chemotherapy-received osteosarcoma and Ewing sarcoma tumors showed no remarkable difference compared to their opposite counterparts (**Figures 2D, E**). The representative images of bone tumor tissues histopathology using hematoxylin and eosin (H&E) staining are illustrated in **Figures 3A–C**, also the images of IGF-1R immunohistochemistry staining with different intensities of immune reactivity are shown in **Figures 3D–I**.

### 3.3 The IGF-1, IGFBP-1 and IGFBP-3 level in osteosarcoma, Ewing sarcoma and GCT

**Figure 4:**
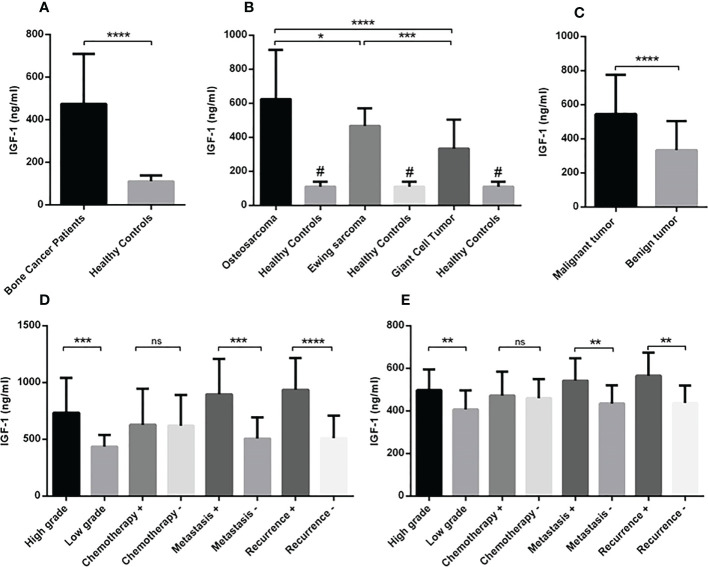
The circulating level of IGF-1 in patients with primary bone tumors. The amount of IGF-1 level in the serum of subjects (patients and healthy controls) was detected using ELISA assay. The increased IGF-1 level was detected in patients with bone tumors (N=90) versus healthy controls (N=30) **(A)**. The osteosarcoma group (N=30) expressed a higher level of IGF-1 compared to the Ewing sarcoma (N=30) and GCT(N=30); while the IGF-1 level was higher in all tumor subtypes compared to the healthy subjects (N=30) **(B)**. Patients with malignant tumors (N=60) showed more IGF-1 levels **(C)** and the high-grade (N=19), metastatic (N=9), and recurrent (N=8) osteosarcoma **(D)** and the high-grade (N=20), metastatic (N=9), and recurrent (N=7) Ewing sarcoma patients showed a higher circulating level of IGF-1 compared to their opposite counterparts. The number of examined samples in each tumor subtype is shown in **Supplementary Table 1**. The statistical differences between groups are shown as asterisks (*P < 0.05, **P <0.01, ***P <0.001, ****P<0.0001), and (ns) indicates unspecific. (#) indicates P<0.0001 for comparing osteosarcoma, Ewing sarcoma, and GCT with healthy subjects.

**Figure 5:**
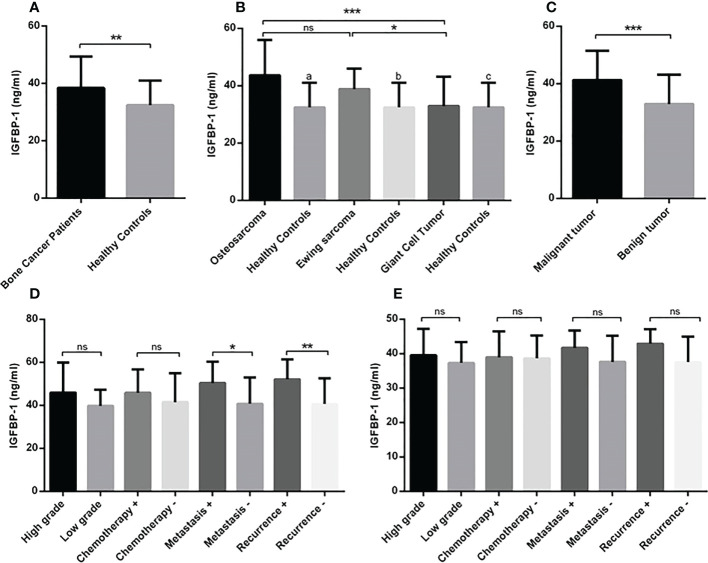
The IGFBP-1 level in patients with primary bone tumors. The IGFBP-1 level is increased in patients with bone tumors (N=90) versus healthy controls (N=30) **(A)**, patients with osteosarcoma (N=30) and Ewing sarcoma (N=30) compared to the healthy controls (N=30) and GCT group (N=30) **(B)**. The patients with malignant tumors (N=60) expressed a higher levels of IGFBP-1 **(C)**; while comparing the tumor subtypes, patients with metastatic (N=9) and recurrent (N=8) osteosarcoma tumors showed higher IGFBP-1 level compared to their opposite counterparts **(D)** and no significant difference was detected in Ewing sarcoma subtypes **(E)**. The number of examined samples in each tumor subtype is shown in **Supplementary Table 1**. The statistical differences between groups are shown as asterisks (*P <0.05, **P <0.01, ***P <0.001), and (ns) indicates unspecific. (a) indicates P=0.0001, (b) indicates P=0.002 and (c) indicated P=0.864 for comparing osteosarcoma, Ewing sarcoma, and GCT with healthy subjects.

**Figure 6:**
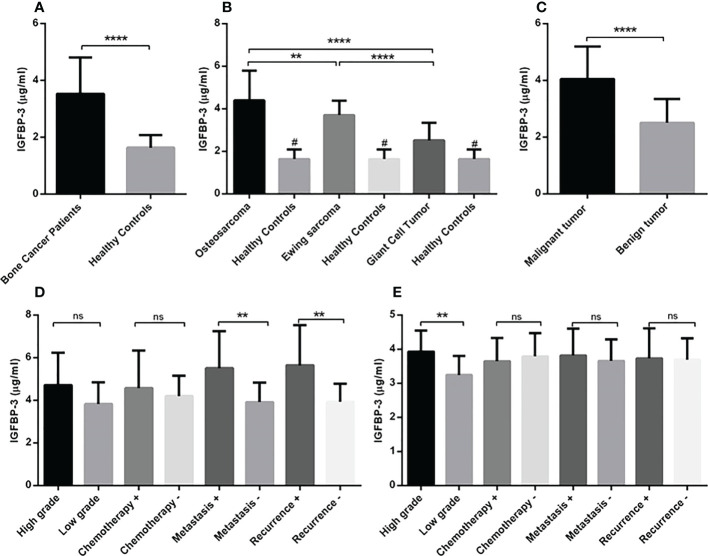
The IGFBP-3 level in patients with primary bone tumors. Patients with bone tumors (N=90) showed a higher level of IGFBP-3 in the serum compared to healthy subjects (N=30) **(A)**. A significant elevation of IGFBP-3 was detected in osteosarcoma (N=30), Ewing sarcoma (N=30) and GCT (N=30) compared to healthy subjects (N=30) also osteosarcoma and Ewing sarcoma group compared to GCT group **(B)**. The IGFBP-3 level in patients with malignant tumors (N=60) was considerable compared to the benign group (N=30) of tumors **(C)**. Patients with metastatic (N=9) and recurrent (N=8) osteosarcoma tumors **(D)** and patients with high-grade (N=20) Ewing sarcoma tumors expressed a higher level of IGFBP-3 compared to their opposite counterparts **(E)**. The number of examined samples in each tumor subtype is shown in **Supplementary Table 1**. The statistical differences between groups are shown as asterisks (**P <0.01, ****P <0.0001), and (ns) indicates unspecific. (#) indicates P<0.0001 for comparing osteosarcoma, Ewing sarcoma, and GCT with healthy subjects.

The circulating level of IGF-1 in serum of patients indicated the elevation of IGF-1 in patients (475.3 ± 24.62) compared to healthy controls (110 ± 5.12) (P < 0.0001) (**Figure 4A**). The IGF-1 level increased significantly in osteosarcoma (624.3 ± 52.92), Ewing sarcoma (467.4 ± 18.80) and GCT (334.2 ± 31.17) groups compared to healthy controls (110.8 ± 28.18) (P < 0.0001); also patients with osteosarcoma produced a significant higher level of IGF-1 compared to patients with Ewing sarcoma (P=0.04) and GCT (P < 0.0001) (**Figure 4B**). The difference of IGF-1level between Ewing sarcoma and GCT was considerable (P < 0.0001) (**Figure 4B**). The IGF-1level between malignant (545.8 ± 29.67) and benign (334.2 ± 31.17) tumors was statistically significant (P < 0.0001) (**Figure 4C**). In addition, the high-grade (733.9 ± 70.71) vs low-grade (435.1 ± 31.20) (P=0.0007), metastatic (897.4 ± 79.2) vs non-metastatic (507.2 ± 40.78) (P=0.0002) and recurrent (937.2 ± 98.38) vs non-recurrent (510.5 ± 42.35) (P < 0.0001) osteosarcoma tumors expressed statistically significant amount of IGF-1 compared to their opposite counterparts (**Figure 4D**). The difference of IGF-1 level between high-grade (497.7 ± 21.73) vs low-grade (406.7 ± 28.47) (P=0.003), metastatic (542.6 ± 34.86) vs non-metastatic (435.1 ± 85.83) (P=0.004) and recurrent (565.9 ± 40.89) vs non-recurrent (437.4 ± 17.14) (P=0.004) Ewing sarcoma tumors was statistically significant (**Figure 4E**). The IGF-1 level showed no remarkable different between chemotherapy-received patients with osteosarcoma and Ewing sarcoma compared to their opposite counterparts. As illustrated in **Figure 5A**, the elevated level of IGFBP-1 was detected in patients with bone tumors (38.47 ± 1.15) compared to healthy subjects (32.48 ± 1.55) (P=0.007). The IGFBP-1 level was increased a significantly in patients with osteosarcoma (43.70 ± 2.23) (P=0.0001) and Ewing sarcoma (38.82 ± 1.29) (P=0.002) compared to the healthy controls (32.48 ± 1.55); while no significant difference was detected between osteosarcoma and Ewing sarcoma. Also, GCT (32.90 ± 1.55) group expressed significantly lower level of IGFBP-1 compared to osteosarcoma (P=0.0005) and Ewing sarcoma (P=0.01) groups (**Figure 5B**). The IGFBP-1 level in serum of patients with malignant tumors (41.26 ± 1.32) was higher compared to patients with benign tumors (32.90 ± 1.86) (P=0.0004) (**Figure 5C**). The patients with metastatic (50.46 ± 3.29) and recurrent (52.11 ± 3.29) osteosarcoma tumors produced a significantly higher level of IGFBP-1 compared to patients with non-metastatic (40.80 ± 2.66) (P=0.04) and non-recurrent (40.64 ± 2.53) (P=0.009) osteosarcoma tumors (**Figure 5D**). The IGFBP-1 level showed no statistically significant difference in patients with different subtypes of Ewing sarcoma (**Figure 5E**). As shown in **Figure 6A**, the IGFBP-3 level was increased significantly in patients with tumors (3.53 ± 0.13) compared to healthy subjects (1.63 ± 0.08) (P < 0.0001). The notable increase in the IGFBP-3 level was detected in patients with osteosarcoma (4.39 ± 0.25), Ewing sarcoma (3.75 ± 0.12) and GCT (2.53 ± 0.15) compared to healthy controls (1.63 ± 0.08) (P < 0.0001); also the difference between patients with osteosarcoma compared to Ewing sarcoma (P=0.003) and GCT (P < 0.0001); and/also Ewing sarcoma and GCT (P < 0.0001) was remarkable (**Figure 6B**). Accordingly, patients with malignant tumors (4.04 ± 0.14) produced higher level of IGFBP-3 in serum compared to patients with benign tumors (2.50 ± 0.15) (P < 0.0001) (**Figure 6C**). The patients with metastatic (5.51 ± 0.57) and recurrent (5.64 ± 0.66) osteosarcoma tumors showed higher level of IGFBP-3 in the serum compared to non-metastatic (3.91 ± 0.20) (P=0.001) and non-recurrent (3.93 ± 0.18) (P=0.001) osteosarcoma tumors (**Figure 6D**). However, between different Ewing sarcoma subtypes, only patients with high-grade tumor (3.93 ± 0.13) expressed a significant elevated level of IGFBP-3 compared to patients with low-grade tumor (3.24 ± 0.17) (P=0.005) (**Figure 6E**).

### 3.4 The association of IGF-1 axis with different demographic features of patients and the diagnostic values

Examining the relationship between levels of IGF-1, IGFBP-1, IGFBP-3, and/also IGF-1R gene and protein expression level with patient age, tumor size, tumor grade, metastasis, tumor recurrence, and malignancy showed the positive correlation between the mentioned tumor features with IGF-1, IGFBP-1, IGFBP-3 and IGF-1R gene and protein expression level. No association was detected regarding the patient’s age and IGF-1 axis that required to be validated by further studies. The IGF-1, IGFBP-3, and IGF-1R gene and protein levels were also significantly correlated with each other; while the IGFBP-1 level in serum correlated significantly just with IGF-1, IGFBP-3, and IGF-1R protein level (The results are shown in **Table 2** and the specificity (p-value) of each factor is illustrated). Additionally, by using a logistic regression model (**Table 3**) and based on IGF-1R protein level, the model could predict significantly the tumor grade (P=0.013), metastasis (P=0.028) and tumor recurrence (P=0.019). Also based on the IGF-1 level and IGF-1R gene expression, the model could predict significantly the tumor grade (P=0.05) and tumor metastasis (P=0.02), respectively. Notably, the diagnostic value of IGF-1, IGFBP-1, IGFBP-3, and/also IGF-1R gene and protein expression in patients with different subtypes of primary bone tumors using ROC curve analysis showed that the IGF-1 levels differed between patients with bone tumors and healthy controls (Cut off > 173.8, AUC=0.98, P<0.0001), patients with a malignant tumor and healthy controls (Cut off > 252.6, AUC=0.99, P<0.0001), patients with benign tumor and healthy controls (Cut off > 173.8, AUC=0.97, P<0.0001) and patients with bone malignant and benign tumor (Cut off > 378.7, AUC=0.81, P<0.0001); IGFBP-1 level differed significantly between patients with bone tumors and healthy controls (Cut off > 33.65, AUC=0.68, P=0.002), patients with a malignant tumor and healthy controls (Cut off > 33.34, AUC=0.76, P<0.0001) and patients with bone malignant and benign tumor (Cut off > 41.25, AUC=0.72, P=0.0005). Accordingly, IGFBP-3 levels differed significantly between patients with bone tumors and healthy controls (Cut off > 2.26, AUC=0.93, P<0.0001), patients with a malignant tumor and healthy controls (Cut off > 2.63, AUC=0.98, P<0.0001), patients with benign tumor and healthy controls (Cut off > 2.26, AUC=0.82, P<0.0001) and patients with bone malignant and benign tumor (Cut off > 3.27, AUC=0.88, P<0.0001); while IGF-1R gene level of expression differed significantly between patients with bone tumors and healthy controls (Cut off > 0.09, AUC=0.72, P<0.0001), patients with a malignant tumor and healthy controls (Cut off > 0.09, AUC=0.83, P<0.0001) and patients with bone malignant and benign tumor (Cut off > 0.06, AUC=0.80, P<0.0001). Also, the IGF-1R protein level of expression differed significantly between patients with bone tumors and healthy controls (Cut off > 10.13, AUC=0.94, P<0.0001), patients with a malignant tumor and healthy controls (Cut off > 15.03, AUC=1, P<0.0001), patients with benign tumor and healthy controls (Cut off > 8.93, AUC=0.83, P<0.0001) and patients with bone malignant and benign tumor (Cut off > 19.98, AUC=1, P<0.0001).

## 4 Discussion

Bone sarcomas, as mesenchymal-originated tumors represent distinct neoplasms with tumoral heterogeneity their prevalence in the pediatric population is significant (17). The variability of response to chemotherapy treatment and lack of appropriate response in some patients is still a noticeable challenge in primary bone tumor therapy (28). Given the dismal prognosis of bone tumors and the inefficiency of current therapies, more effective treatment strategies and efficient diagnostic methods are urgently required. There is increasing evidence suggesting that the IGF-1 receptor and related mediators promote the growth, metabolism, and differentiation of cancer cells (29). The multifactorial IGF-1 family consists of IGF ligands, receptor and binding proteins that exhibits aberrant expression in favor of cancer genesis in various tumor types (2). According to the cooperative role of the IGF family in bone homeostasis and regulatory effects in bone length and growth, the hypothesis of their influence on the pathogenesis of bone tumors becomes stronger (10). Examining the IGF-1 family revealed an increase in the gene and protein level of IGF-1R in bone tumors of osteosarcoma and Ewing sarcoma compared to tumor margins as well as the circulating level of IGF-1, IGFBP-, and IGFBP-3 in these patients. The association of the IGF-1 axis with the severity of the tumor (grade and size) and tumor metastasis and recurrence indicates its possible role in the progression of osteosarcoma and Ewing sarcoma. In support of our data, the elevated level of IGF-1R mRNA and protein in osteosarcoma tissue and its correlation with tumor stage and distant metastasis was reported (30). Moreover, the local expression of IGF-1R (31), and the circulating expression of IGF-1 and IGFBP-3 (32) were detected in patients with Ewing sarcoma. Based on our data, no noticeable changes in the IGF-1R gene and protein, but a relative increase in circulating levels of IGF-1, IGFBP-1, and 3 were detected in GCT tissues and serum. Comparing the osteosarcoma, Ewing sarcoma, and GCT groups, the over-expression of the IGF-1 axis was more prominent in osteosarcoma and Ewing sarcoma compared to GCT which might be influenced by higher tumor grades and disease stages in these groups. It was shown that the expression level of IGF-1 in recurrent GCT was significantly higher than in non-recurrent GCT subjects (33); however, GCT cases in our study were GCT patients without recurrence. Most of the studies in the literature regarding the IGF-1 axis in bone tumor pathogenesis are focused on determining the functional mechanism of this axis. Evidence revealed that IGF-1R downstream signaling through MEK/ERK pathway (34) and Akt activation mediates migration and invasion of osteosarcoma cells (30). Interestingly, IGF-1 stimulates collagen-1 production in osteosarcoma cells that can further arrange cells, shape, behavior, and proliferation (35). Another study showed that IGFBP-3 induced the expression of VCAM-1 and mediates the migration of human osteosarcoma cells through the activation of PI3K, Akt, and AP-1 signaling pathways (36). It seemed that the effect of the IGF-1 axis on the expression or activity of extracellular matrix (ECM) components facilitates bone tumor cell promotion. Accordingly, blockage of IGF-1R caused down-regulation of matrix metalloproteinase-2 (MMP-2) and MMP-9 and suppress migration of human osteosarcoma (OS) MG-63 cells (37). In support of this, our previously published data showed the over-expression of MMP-9 and its association with tumor recurrence and metastasis of osteosarcoma and Ewing sarcoma (21). The simultaneous increase of IGF-1R and its associated mediators in the same set of samples in our current study can confirm the possible role of the IGF-1 axis on the change of ECM proteases in favor of bone cancer cell invasion. Moreover, the effect of exogenous biglycan on the activation of IGF-1R and the mediatory role of β-catenin hypothesize the plausible effect of β-catenin/IGF-1R signaling pathway in controlling the osteosarcoma cell growth (38). Notably, β-catenin showed clinical significance and positive association with primary bone tumor aggressiveness in our previous study which can emphasize the possible relation of IGF-1R and β-catenin in bone tumor pathogenesis (22). Regarding the stimulating or inhibiting role of the IGF-1 axis in the pathogenesis of Ewing sarcoma, the evidence is conflicting. It was shown that the high level of IGF-1 in the serum of patients with Ewing sarcoma was correlated with a lower risk of tumor progression (31). In addition, the improved overall survival (OS) and event-free survival of patients with Ewing sarcoma were correlated with an elevated level of IGF-1 and IGFBP3 (32). Also, it was illustrated that exogenous IGFBP3 reduced Ewing sarcoma motility and growth (39). Data from the current study showed over-expression of IGF-1R, IGF-1, IGFBP-1, and 3 in local tumor tissues and serum of patients with Ewing sarcoma. Although the circulating level of IGFBP-1 and 3 showed no significant correlation with tumor metastasis and recurrence in Ewing sarcoma; this correlation was significant for tumor relapse and metastasis of osteosarcoma patients. The observed difference may be due to the different etiology of Ewing sarcoma regarding the specific chromosomal translocation t (11, 22)(q24;q12) in these tumors that is required for encoding the fusion protein, EWS::FLI1, which is a modifier and acts as a master regulator of Ewing sarcoma (40). It was shown that inhibition of EWS::FLI1protein was associated with reduced IGF-1/IGF-1R signaling and induced Ewing sarcoma apoptosis and death that is postulated to be related to the interaction of IGF-1R and focal adhesion kinase (FAK) (41). Investigating the relevance of IGF-1R over-expression with poor outcomes and survival of Ewing sarcoma remained inconclusive (42) and it seemed that contradictions need to be verified by future mechanistic surveys. In the current study the diagnostic value of the IGF-1 axis to discriminate patients with osteosarcoma and Ewing sarcoma from healthy subjects was considerable; to propose this axis as a diagnostic marker, more samples need to be examined.

Ultimately, it should be noted that this study had some limitations. First, the relationship between the age of the patients and the level of the IGF-1 axis in this study was inconclusive. Considering that IGF-1 secretion occures in an age-dependent manner and its level is increased during puberty (43), it is possible that an unequal distribution of patients in terms of age in this study may lead to the lack of appropriate conclusions in this field and investigating the relationship between the age of patients with bone tumors and the level of IGF axis production should be perused in future studies. Second, the relationship between the effect of receiving chemotherapy treatment on the level of IGF-1 axis in patients with malignant bone tumors was inconclusive, and investigating the relationship between receiving chemotherapy treatment and the rate of response to treatment in patients as well as changes in tumor histology on circulating and local IGF-1 axis levels should be investigated in future studies.

## 5 Conclusion

Due to the comprehensive investigation of the local and circulating expression profile of the important effectors of the IGF-1 axis in three types of common bone tumors, our study can provide important evidence for the more effective design of chemotherapy treatments based on the inhibitors of this axis. Over-expression of the IGF-1 axis may account for an as negative prognostic biomarker for patients suffering from primary bone cancers especially osteosarcoma.

## Data availability statement

The original contributions presented in the study are included in the article/**Supplementary Material**. Further inquiries can be directed to the corresponding author.

## Ethics statement

The studies involving human participants were reviewed and approved by Iran University of Medical Sciences with ethics committee code: IR.IUMS.FMD.REC.1400.393. Written informed consent to participate in this study was provided by the participants’ legal guardian/next of kin.

## Author contributions

MV performed the experiments, data collection and analysis. AE, BS, SY, and FA-F contributed in data analysis. PB, AM and MR provided samples and performed immunohistochemistry. VS and MT-Y designed and conceived the study and wrote the manuscript. All authors read and approved the final manuscript

## References

[1] Dehkhoda F Lee CMM Medina J Brooks AJ. The growth hormone receptor: Mechanism of receptor activation, cell signaling, and physiological aspects. Front endocrinol (2018) 9:35. doi: 10.3389/fendo.2018.00035 PMC5816795 29487568

[2] Hakuno F Takahashi SI. IGF1 receptor signaling pathways. J Mol Endocrinol (2018) 61(1):T69–t86. doi: 10.1530/JME-17-0311 29535161

[3] Blum WF Alherbish A Alsagheir A El Awwa A Kaplan W Koledova E. The growth hormone-insulin-like growth factor-I axis in the diagnosis and treatment of growth disorders. Endocr connect (2018) 7(6):R212–r22. doi: 10.1530/EC-18-0099 PMC5987361 29724795

[4] Allard JB Duan C. IGF-binding proteins: Why do they exist and why are there so many? Front Endocrinol (2018) 9:117. doi: 10.3389/fendo.2018.00117 PMC5900387 29686648

[5] Kasprzak A. Insulin-like growth factor 1 (IGF-1) signaling in glucose metabolism in colorectal cancer. Int J Mol Sci (2021) 22(12):6434. doi: 10.3390/ijms22126434 34208601 PMC8234711

[6] Anisimov VN Bartke A. The key role of growth hormone-insulin-IGF-1 signaling in aging and cancer. Crit Rev oncol/hematol (2013) 87(3):201–23. doi: 10.1016/j.critrevonc.2013.01.005 PMC4095988 23434537

[7] Samani AA Yakar S LeRoith D Brodt P. The role of the IGF system in cancer growth and metastasis: overview and recent insights. Endocr Rev (2007) 28(1):20–47. doi: 10.1210/er.2006-0001 16931767

[8] Chen PN Lin CW Yang SF Chang YC. Oral submucous fibrosis stimulates invasion and epithelial-mesenchymal transition in oral squamous cell carcinoma by activating MMP-2 and IGF-IR. J Cell Mol Med (2021) 25(20):9814–25. doi: 10.1111/jcmm.16929 PMC8505822 34528373

[9] Armakolas N Armakolas A Antonopoulos A Dimakakos A Stathaki M Koutsilieris M. The role of the IGF-1 ec in myoskeletal system and osteosarcoma pathophysiology. Crit Rev oncol/hematol (2016) 108:137–45. doi: 10.1016/j.critrevonc.2016.11.004 27931832

[10] Yakar S Werner H Rosen CJ. Insulin-like growth factors: actions on the skeleton. J Mol Endocrinol (2018) 61(1):T115–t37. doi: 10.1530/JME-17-0298 PMC5966339 29626053

[11] Hernández-Sánchez C Blakesley V Kalebic T Helman L LeRoith D. The role of the tyrosine kinase domain of the insulin-like growth factor-I receptor in intracellular signaling, cellular proliferation, and tumorigenesis. J Biol Chem (1995) 270(49):29176–81. doi: 10.1074/jbc.270.49.29176 7493944

[12] Guntur AR Rosen CJ. IGF-1 regulation of key signaling pathways in bone. BoneKEy Rep (2013) 2:437. doi: 10.1038/bonekey.2013.171 24422135 PMC3818534

[13] Ambrogini E Almeida M Martin-Millan M Paik JH Depinho RA Han L. FoxO-mediated defense against oxidative stress in osteoblasts is indispensable for skeletal homeostasis in mice. Cell Metab (2010) 11(2):136–46. doi: 10.1016/j.cmet.2009.12.009 PMC2819984 20142101

[14] Laplante M Sabatini DM. Regulation of mTORC1 and its impact on gene expression at a glance. J Cell Sci (2013) 126(Pt 8):1713–9. doi: 10.1242/jcs.125773 PMC3678406 23641065

[15] Itoh S Saito T Hirata M Ushita M Ikeda T Woodgett JR. GSK-3α and GSK-3β proteins are involved in early stages of chondrocyte differentiation with functional redundancy through RelA protein phosphorylation. J Biol Chem (2012) 287(35):29227–36. doi: 10.1074/jbc.M112.372086 PMC3436165 22761446

[16] Bachrach LK. Hormonal contraception and bone health in adolescents. Front endocrinol (2020) 11:603. doi: 10.3389/fendo.2020.00603 PMC7472551 32973688

[17] Brown HK Schiavone K Gouin F Heymann MF Heymann D. Biology of bone sarcomas and new therapeutic developments. Calcif Tissue Int (2018) 102(2):174–95. doi: 10.1007/s00223-017-0372-2 PMC5805807 29238848

[18] Hosseini A Mirzaei A Salimi V Jamshidi K Babaheidarian P Fallah S. The local and circulating SOX9 as a potential biomarker for the diagnosis of primary bone cancer. J Bone Oncol (2020) 23:100300. doi: 10.1016/j.jbo.2020.100300 32551218 PMC7292907

[19] Viereck V Siggelkow H Pannem R Braulke T Scharf JG Kübler B. Alteration of the insulin-like growth factor axis during *in vitro* differentiation of the human osteosarcoma cell line HOS 58. J Cell Biochem (2007) 102(1):28–40. doi: 10.1002/jcb.21274 17372931

[20] Dale O Salo M. The Helsinki declaration, research guidelines and regulations: present and future editorial aspects. Acta anaesthesiol Scand (1996) 40(7):771–2. doi: 10.1111/j.1399-6576.1996.tb04530.x 8874560

[21] Vaezi MA Eghtedari AR Safizadeh B Ghasempour G Salimi V Nourbakhsh M. Up-regulation of matrix metalloproteinase-9 in primary bone tumors and its association with tumor aggressiveness. Mol Biol Rep (2022) 49(10):9409–27. doi: 10.1007/s11033-022-07798-z 36002655

[22] Khademian N Mirzaei A Hosseini A Zare L Nazem S Babaheidarian P. Expression pattern and clinical significance of β-catenin gene and protein in patients with primary malignant and benign bone tumors. Sci Rep (2022) 12(1):9488. doi: 10.1038/s41598-022-13685-1 35676319 PMC9177768

[23] Heck RK Jr. Peabody TD Simon MA. Staging of primary malignancies of bone. CA: Cancer J Clin (2006) 56(6):366–75. doi: 10.3322/canjclin.56.6.366 17135693

[24] Casali PG Bielack S Abecassis N Aro HT Bauer S Biagini R. Bone sarcomas: ESMO-PaedCan-EURACAN clinical practice guidelines for diagnosis, treatment and follow-up. Ann Oncol (2018) 29(Suppl 4):iv79–95. doi: 10.1093/annonc/mdy310 30285218

[25] Crowe AR Yue W. Semi-quantitative determination of protein expression using immunohistochemistry staining and analysis: An integrated protocol. Bio-protocol (2019) 9(24):e3465. doi: 10.21769/BioProtoc.3465 31867411 PMC6924920

[26] Eghtedari AR Vaezi MA Safizadeh B Ghasempour G Babaheidarian P Salimi V. Evaluation of the expression pattern and diagnostic value of PPARγ in malignant and benign primary bone tumors. BMC musculoskeletal Disord (2022) 23(1):746. doi: 10.1186/s12891-022-05681-3 PMC9347110 35922782

[27] Hajian-Tilaki K. Receiver operating characteristic (ROC) curve analysis for medical diagnostic test evaluation. Caspian J Internal Med (2013) 4(2):627–35. PMC3755824 24009950

[28] Isakoff MS Bielack SS Meltzer P Gorlick R. Osteosarcoma: Current treatment and a collaborative pathway to success. J Clin Oncol (2015) 33(27):3029–35. doi: 10.1200/JCO.2014.59.4895 PMC4979196 26304877

[29] Mancarella C Morrione A Scotlandi K. Novel regulators of the IGF system in cancer. Biomolecules (2021) 11(2):273. doi: 10.3390/biom11020273 33673232 PMC7918569

[30] Wang YH Han XD Qiu Y Xiong J Yu Y Wang B. Increased expression of insulin-like growth factor-1 receptor is correlated with tumor metastasis and prognosis in patients with osteosarcoma. J Surg Oncol (2012) 105(3):235–43. doi: 10.1002/jso.22077 21866554

[31] Scotlandi K Manara MC Serra M Marino MT Ventura S Garofalo C. Expression of insulin-like growth factor system components in ewing's sarcoma and their association with survival. Eur J Cancer (Oxford Engl 1990) (2011) 47(8):1258–66. doi: 10.1016/j.ejca.2011.01.007 21345666

[32] de Groot S Gelderblom H Fiocco M Bovée JV van der Hoeven JJ Pijl H. Serum levels of IGF-1 and IGF-BP3 are associated with event-free survival in adult Ewing sarcoma patients treated with chemotherapy. OncoTargets Ther (2017) 10:2963–70. doi: 10.2147/OTT.S123726 PMC5476720 28652778

[33] Chen S Du Z Wu B Shen H Liu C Qiu X. STAT1, IGF1, RAC1, and MDM2 are associated with recurrence of giant cell tumor of bone. J Immunol Res (2018) 2018:4564328. doi: 10.1155/2018/4564328 29651441 PMC5831922

[34] Yu Y Luk F Yang JL Walsh WR. Ras/Raf/MEK/ERK pathway is associated with lung metastasis of osteosarcoma in an orthotopic mouse model. Anticancer Res (2011) 31(4):1147–52. 21508358

[35] Nasu M Sugimoto T Kaji H Chihara K. Estrogen modulates osteoblast proliferation and function regulated by parathyroid hormone in osteoblastic SaOS-2 cells: role of insulin-like growth factor (IGF)-I and IGF-binding protein-5. J endocrinol (2000) 167(2):305–13. doi: 10.1677/joe.0.1670305 11054645

[36] Chao CC Lee WF Yang WH Lin CY Han CK Huang YL. IGFBP-3 stimulates human osteosarcoma cell migration by upregulating VCAM-1 expression. Life Sci (2021) 265:118758. doi: 10.1016/j.lfs.2020.118758 33188835

[37] Lin F Shen Z Xu X Hu BB Meerani S Tang LN. Evaluation of the expression and role of IGF pathway biomarkers in human sarcomas. Int J immunopathol Pharmacol (2013) 26(1):169–77. doi: 10.1177/039463201302600116 23527719

[38] Aggelidakis J Berdiaki A Nikitovic D Papoutsidakis A Papachristou DJ Tsatsakis AM. Biglycan regulates MG63 osteosarcoma cell growth through a LPR6/β-Catenin/IGFR-IR signaling axis. Front Oncol (2018) 8:470. doi: 10.3389/fonc.2018.00470 30406034 PMC6206209

[39] Benini S Zuntini M Manara MC Cohen P Nicoletti G Nanni P. Insulin-like growth factor binding protein 3 as an anticancer molecule in ewing's sarcoma. Int J cancer (2006) 119(5):1039–46. doi: 10.1002/ijc.21929 16570284

[40] Ahmed NS Harrell LM Wieland DR Lay MA Thompson VF Schwartz JC. Fusion protein EWS-FLI1 is incorporated into a protein granule in cells. RNA (New York NY) (2021) 27(8):920–32. doi: 10.1261/rna.078827.121 PMC8284321 34035145

[41] Moritake H Saito Y Sawa D Sameshima N Yamada A Kinoshita M. TAE226, a dual inhibitor of focal adhesion kinase and insulin-like growth factor-I receptor, is effective for Ewing sarcoma. Cancer Med (2019) 8(18):7809–21. doi: 10.1002/cam4.2647 PMC6912025 31692287

[42] Liang J Li B Yuan L Ye Z. Prognostic value of IGF-1R expression in bone and soft tissue sarcomas: a meta-analysis. OncoTargets Ther (2015) 8:1949–55. doi: 10.2147/OTT.S88293 PMC4524581 26251617

[43] Olarescu NC Gunawardane K Hansen TK Møller N Jørgensen JOL. Normal physiology of growth hormone in adults. In: Feingold KR Anawalt B Boyce A Chrousos G de Herder WW Dhatariya K, editors. Endotext. (South Dartmouth, MA: MDText.com, Inc.) (2000).

