# Supplementary material for: Evaluating the local expression pattern of IGF-1R in tumor tissues and the circulating levels of IGF-1, IGFBP-1, and IGFBP-3 in the blood of patients with different primary bone tumors
Source: Front Oncol. 2023 Jan 13;12:1096438. doi: 10.3389/fonc.2022.1096438 (PMC9880312; doi:10.3389/fonc.2022.1096438)
Supplement: Supplementary file 1 [file DataSheet_1.docx]

**Supplementary table 1: The association of IGF-1, IGFBP-1, IGFBP-3 and IGF-1R with bone cancer different features**

| Variables | Subgroups | Osteosarcoma (n=30) | | Ewing sarcoma (n=30) | | Giant Cell Tumor (n=30) | | Healthy individuals | |
| --- | --- | --- | --- | --- | --- | --- | --- | --- | --- |
|  |  | Number | Percent | Number | Percent | Number | Percent | Number | Percent |
| Age | 15-20 | 4 | 13.33% | 11 | 36.66% | 8 | 26.66% | 5 | 16.66% |
|  | 20-30 | 7 | 23.33% | 9 | 30% | 8 | 26.66% | 10 | 33.33% |
|  | ≥30 | 19 | 63.33% | 10 | 33.33% | 14 | 46.66% | 15 | 50% |
| Gender | Male | 16 | 53.3% | 12 | 40% | 17 | 56.7% | 15 | 50% |
|  | Female | 14 | 46.7% | 18 | 60% | 13 | 43.3% | 15 | 50% |
| Tumor grade | Low | 11 | 36.7% | 10 | 33.3% | 30 | 100% | - | - |
|  | High | 19 | 63.3% | 20 | 66.7% | 0 | 0 | - | - |
| Metastasis | Positive | 9 | 30% | 9 | 30% | 0 | 0 | - | - |
|  | Negative | 21 | 70% | 21 | 70% | 30 | 100% | - | - |
| Chemotherapy | Received | 15 | 50% | 18 | 60% | 0 | 0 | - | - |
|  | Not received | 15 | 50% | 12 | 40% | 30 | 100% | - | - |
| Recurrence | Positive | 8 | 26.7% | 7 | 23.3% | 0 | 0 | - | - |
|  | Negative | 22 | 73.33% | 23 | 76.7% | 30 | 100% | - | - |
| Tumor size | 10 cm > | 16 | 53.3% | 18 | 60% | 26 | 86.7% | - | - |
|  | ≥10 cm | 14 | 46.7% | 12 | 40% | 4 | 13.3% | - | - |

**Supplementary table 2: Group and intergroup comparison of IGF-1R gene expression levels in primary bone tumor tissues**

| Mann–Whitney test | Groups | Minimum | Median | Maximum | Summary | Individual P value |
| --- | --- | --- | --- | --- | --- | --- |
| Tumor tissue vs. Normal margin | Tumor tissue | 0.001246 | 0.1510 | 0.7642 | **** | P < 0.0001 |
|  | Normal margin | 0.0001853 | 0.02900 | 0.5872 |  |  |
| Osteosarcoma vs. Normal margin | Osteosarcoma | 0.007099 | 0.2391 | 0.7642 | **** | P < 0.0001 |
|  | Normal margin | 0.0007943 | 0.02936 | 0.4419 |  |  |
| Ewing sarcoma vs. Normal margin | Ewing sarcoma | 0.01372 | 0.1565 | 0.7280 | **** | P < 0.0001 |
|  | Normal margin | 0.0001853 | 0.02900 | 0.5555 |  |  |
| Giant Cell Tumor vs. Normal margin | Giant Cell Tumor | 0.001246 | 0.03356 | 0.3169 | ns | 0.8804 |
|  | Normal margin | 0.0005616 | 0.03004 | 0.5872 |  |  |
| Osteosarcoma vs. Ewing sarcoma | Osteosarcoma | 0.007099 | 0.2391 | 0.7642 | ns | 0.2286 |
|  | Ewing sarcoma | 0.01372 | 0.1565 | 0.7280 |  |  |
| Osteosarcoma vs. Giant Cell Tumor | Osteosarcoma | 0.007099 | 0.2391 | 0.7642 | **** | P < 0.0001 |
|  | Giant Cell Tumor | 0.001246 | 0.03356 | 0.3169 |  |  |
| Ewing sarcoma vs. Giant Cell Tumor | Ewing sarcoma | 0.01372 | 0.1565 | 0.7280 | **** | P < 0.0001 |
|  | Giant Cell Tumor | 0.001246 | 0.03356 | 0.3169 |  |  |
| Malignant tumor vs. Benign tumor | Malignant tumor | 0.007099 | 0.1999 | 0.7642 | **** | P < 0.0001 |
|  | Benign tumor | 0.001246 | 0.03356 | 0.3169 |  |  |
| Osteosarcoma High grade vs. Low grade tumors | High grade | 0.02237 | 0.2556 | 0.7642 | ns | 0.6338 |
|  | Low grade | 0.007099 | 0.2225 | 0.4239 |  |  |
| Osteosarcoma Chemotherapy+ vs. Chemotherapy- tumors | Chemotherapy+ | 0.007099 | 0.2225 | 0.6935 | ns | 0.5194 |
|  | Chemotherapy- | 0.04304 | 0.2864 | 0.7642 |  |  |
| Osteosarcoma metastasis+ vs. metastasis - tumors | metastasis+ | 0.2105 | 0.5007 | 0.7642 | **** | P < 0.0001 |
|  | metastasis - | 0.007099 | 0.1618 | 0.4239 |  |  |
| Osteosarcoma recurrence+ vs. recurrence - | recurrence+ | 0.04304 | 0.2710 | 0.6935 | ns | 0.4411 |
|  | recurrence - | 0.007099 | 0.2088 | 0.7642 |  |  |
| Ewing sarcoma High grade vs. Low grade tumors | High grade | 0.01372 | 0.1492 | 0.7280 | ns | 0.9564 |
|  | Low grade | 0.06479 | 0.1714 | 0.7280 |  |  |
| Ewing sarcoma Chemotherapy+ vs. Chemotherapy- tumors | Chemotherapy+ | 0.01372 | 0.1850 | 0.7280 | ns | 0.2355 |
|  | Chemotherapy- | 0.06479 | 0.1193 | 0.5555 |  |  |
| Ewing sarcoma metastasis+ vs. metastasis - tumors | metastasis+ | 0.1201 | 0.3280 | 0.7280 | * | 0.05 |
|  | metastasis - | 0.01372 | 0.1168 | 0.2469 |  |  |
| Ewing sarcoma recurrence+ vs. recurrence - | recurrence+ | 0.1201 | 0.3280 | 0.7280 | *** | 0.0009 |
|  | recurrence - | 0.01372 | 0.1234 | 0.5555 |  |  |
